# Ginsenoside Rg2 Attenuated Trastuzumab-Induced Cardiotoxicity in Rats

**DOI:** 10.1155/2022/8866660

**Published:** 2022-01-12

**Authors:** Guang Liu, Jinli Zhang, Fangyi Sun, Jingtao Ma, Xiaoyong Qi

**Affiliations:** ^1^Graduate School of Hebei Medical University, 361 East Zhongshan Road, Shijiazhuang, Hebei 050000, China; ^2^Cardiovascular Department of the Fourth Hospital of Hebei Medical University, 12 Jiankang Road, Shijiazhuang, Hebei 050000, China; ^3^Cardiology Center, Hebei Provincial People's Hospital, 348 West Heping Road, Shijiazhuang, Hebei 050000, China

## Abstract

**Aim:**

Trastuzumab (TZM) is a monoclonal antibody drug for HER2-positive breast cancer by targeting epidermal growth factor 2, but it has significant cardiotoxicity. Ginsenoside Rg2 has shown a variety of biological activities. This study was aimed at investigating whether Rg2 attenuates TZM-induced cardiotoxicity.

**Methods:**

A model of TZM-induced cardiotoxicity was established in Wistar rats, and the rats were pretreated with Rg2. After echocardiography analysis, the rats were killed and the hearts were dissected for RNAseq analysis. Primary human cardiomyocytes (HCMs) were treated with TZM with or without pretreatment with Rg2 and then subjected to a colony formation assay, flow cytometry analysis, and Western blot analysis for the detection of caspase-3, caspase-9, and BAX.

**Results:**

TZM induced LV dysfunction in rats, but Rg2 could attenuate TZM-induced LV dysfunction. The mRNA levels of caspase-3, caspase-9, and BAX were significantly higher in TZM-treated rats. The colony formation ability of HCMs was significantly lower in TZM-treated cells but was recovered after pretreatment with Rg2. The apoptosis rate of HCMs was significantly higher in TZM-treated cells but was significantly lower after pretreatment with Rg2. Moreover, protein levels of caspase-3, caspase-9, and BAX were significantly higher in TZM-treated cells but were significantly lower after pretreatment with Rg2.

**Conclusion:**

Ginsenoside Rg2 inhibited TZM-induced cardiotoxicity, and the mechanism may be related to the downregulation of the expression of proapoptotic proteins caspase-3, caspase-9, and BAX and the inhibition of TZM-induced apoptosis in cardiomyocytes. Ginsenoside Rg2 has a potential to be applied in patients with breast cancer to prevent TZM-induced cardiotoxicity.

## 1. Introduction

Trastuzumab (TZM) is a humanized monoclonal antibody used as a drug to target the extracellular domain of human epidermal growth factor receptor 2 (HER2) and has shown good therapy efficacy on the majority of patients with HER2-positive breast cancer [1]. In particular, recent efforts have been focused on the development of antibody-drug conjugates of TZM with high affinity, specificity, and efficacy for the treatment of HER2-positive breast cancer [ 2, 3]. However, TZM causes several side effects on the heart known as cardiotoxicity, including hypertension, QT prolongation and bradycardia, congestive heart failure, and ischemic heart disease [4, 5]. Therefore, it is urgent to limit the cardiotoxicity of TZM to achieve maximum efficacy on breast cancer therapy.

Ginseng is a traditional Chinese medicine widely used in East Asia countries such as China, Bhutan, and Korea [6]. Ginsenoside Rg2 is one of the main compounds of ginseng that exert drug efficacy [7]. Ginsenoside Rg2 has shown a variety of biological activities such as enhancing memory, improving metabolism, and protecting the heart [8–10]. Therefore, we wondered whether ginsenoside Rg2 could reduce TZM-induced cardiotoxicity. This study was aimed at establishing an animal model of TZM-induced cardiotoxicity and investigating the mechanism by which ginsenoside Rg2 attenuates TZM-induced cardiotoxicity.

## 2. Materials and Methods

### 2.1. Animals

All animal protocols were approved by the Animal Care and Use Committee of Hebei Medical University. Specific pathogen-free Wistar rats (male, 6 weeks old) were purchased from Charles River Laboratories and kept in a room with controlled temperature at 25°C and a 12 h light/dark cycle.

### 2.2. Experimental Groups

The rats were randomly divided into three groups (*n* = 10). In the control group, rats were injected intraperitoneally with saline. In the T group, rats were injected intraperitoneally with TZM (Roche Diagnostics) for seven days (the dose at the first day was 12 mg/kg/day, and the dose at the following days was 6 mg/kg/day). In the T+Rg2 group, rats were injected intraperitoneally with Rg2 (purity > 98%, Sigma) at the dose of 15 mg/kg one day before the injection of TZM and then injected intraperitoneally with TZM in the same manner as the T group. The cardiac tissues were collected from the greater curvature of the stomach after the rats were euthanized with sodium pentobarbital (dose > 50 mg/kg).

### 2.3. Echocardiography

Rats were lightly anesthetized with 1% isoflurane to maintain the heart rate at 350 bpm. Heart function was monitored by echocardiography using a VEVO 2100 high-resolution imaging system (VisualSonics). Two-dimensional short-axis M-mode echocardiography was performed to measure left ventricular end diastolic diameter (LVEDD), left ventricular end systolic diameter (LVESD), interventricular septal thickness in diastolic end (IVSTd), interventricular septal thickness in systolic end (LVSTs), left ventricular posterior wall thickness in diastolic end (LVPWd), left ventricular posterior wall thickness in systolic end (LVPWs), ejection fraction (EF), fractional shortening (FS), left ventricular systolic pressure (LVSP), and left ventricular end-diastolic pressure (LVEDP), as described previously [11].

### 2.4. RNAseq

Rats were humanly euthanized by cervical dislocation. The hearts were dissected, washed with phosphate-buffered saline (PBS), and resuspended in the TRIzol reagent (Takara, Tokyo, Japan). Next, the hearts were homogenized using a polytron homogenizer, and total RNA was extracted from homogenized heart samples following the protocols of the TRIzol reagent. The purified RNA samples were sent to Novartis (Basel, Switzerland) for next-generation sequencing.

### 2.5. Cell Culture

Primary human cardiomyocytes (HCMs) were purchased from Applied Biological and cultured in DMEM (Sigma-Aldrich) supplemented with 10% fetal bovine serum (FBS, Thermo Fisher Scientific, Inc.) and 1% streptomycin-penicillin at 37°C in atmosphere with 5% CO_2_. When cells grew to 80% confluency, HCMs were pretreated with 200 *μ*M ginsenoside Rg2 at 37°C for 12 h and then treated with 100 *μ*g/mL TZM at 37°C for 24 h.

### 2.6. Clone Formation Assay

HCMs were seeded at a density of 500 cells/well in 6-well plates and were cultured in DMEM supplemented with 10% FBS and 1% streptomycin-penicillin at 37°C in atmosphere with 5% CO_2_. The medium was changed every 2–3 days. After culture for 8 days, colonies were rinsed with phosphate-buffered saline (PBS), fixed in 2% paraformaldehyde, and stained with 0.05% (*v*/*v*) crystal violet.

### 2.7. Flow Cytometry

The apoptosis of HCMs was examined by using an Annexin V/7 AAD apoptosis kit (BD Biosciences, USA). Briefly, HCMs were collected, washed with PBS, and resuspended in 100 *μ*L binding buffer. Then, the cells were incubated with Annexin V and 7-AAD in the dark at 4°C for 1 h. The stained cells were washed with PBS and analyzed using the FACSCalibur System (Becton-Dickinson) for flow cytometry. The proportion of positively stained cells was calculated to determine the apoptosis ratio.

### 2.8. Western Blot Analysis

HCMs were washed with PBS and lysed in RIPA lysis buffer supplemented with a protease inhibitor. Cell lysates were centrifuged at 10,000 × *g* for 10 min at 4°C. The protein concentrations were determined by using a bicinchoninic acid protein quantification kit (Promega). Equal amounts of proteins were separated by 10% SDS-PAGE and transferred onto PVDF membranes (Millipore). The membranes were blocked with 5% nonfat milk for 1 h at room temperature. The membranes were then incubated with antibodies for caspase-3, caspase-9, BAX, and *β*-actin (all from Abcam) at 4°C overnight. Next, the membranes were washed with PBS and incubated with horseradish peroxidase-conjugated secondary antibodies (Abcam) at room temperature for 1 h. Immunoreactive bands were detected with the ECL reagent (Santa Cruz Biotechnology, Inc.), and the densities were quantified by using ImageJ software (National Institutes of Health).

### 2.9. Statistical Analysis

Statistical analysis was performed using SPSS software version 17.0 (SPSS, Inc.). All results were shown as mean ± standard deviation (SD). Student's *t*-test was used to compare differences in two groups. One-way and two-way ANOVA were used to compare differences among multiple groups, followed by post hoc Bonferroni's test. Statistical significance was defined at *P* < 0.05.

## 3. Results

### 3.1. Rg2 Attenuated TZM-Induced LV Dysfunction

**Figure 1:**
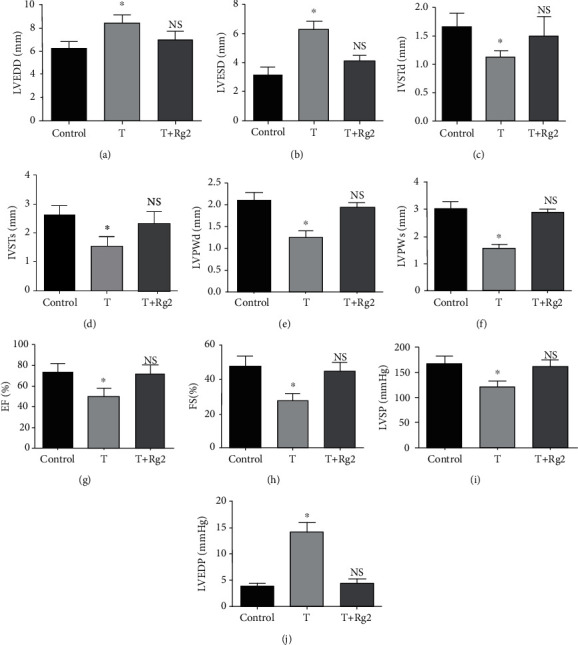
Echocardiographic analysis of rats in three groups: (a) LVEDD; (b) LVESD; (c) IVSTd; (d) IVSTs; (e) LVPWd; (f) LVPWs; (g) EF; (h) FS; (i) LVSP; (j) LVEDP. Data were shown as mean ± SD (*n* = 10). ^∗^*P* < 0.05 compared to other groups.

First, we performed echocardiographic analysis on three groups of rats. The results showed that LVEDD, LVESD, and LVEDP were significantly higher while IVSTd, IVSTs, LVPWd, LVPWs, EF, FS, and LVSP were significantly lower in the T group than in the control group. However, all these parameters showed no significant differences between the T+Rg2 group and control group (Figures 1(a)–1(j)). Collectively, these data suggest that TZM induces LV dysfunction in rats, but Rg2 could attenuate TZM-induced LV dysfunction.

### 3.2. Enrichment of Apoptosis-Related Genes in the Hearts of Rats Treated with TZM

**Figure 2:**
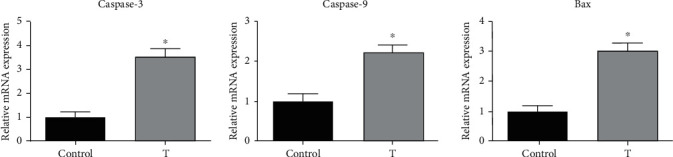
The mRNA levels of caspase-3, caspase-9, and BAX in the hearts of rats in three groups. Data were shown as mean ± SD (*n* = 3). ^∗^*P* < 0.05 compared to the control group.

To reveal the molecular mechanism by which TZM induced LV dysfunction, we performed RNAseq analysis of the hearts of control and T groups. Comparison of two groups showed that caspase-3, caspase-9, and BAX were three genes significantly upregulated in the T group. To confirm the results, we performed PCR analysis and found that relative mRNA levels of caspase-3, caspase-9, and BAX were significantly higher in the T group than in the control group (Figure 2). These results suggest that TZM-induced LV dysfunction is related to the promotion of apoptosis in the heart.

### 3.3. Rg2 Promoted Colony Formation of HCMs and Inhibited TZM-Induced Apoptosis in HCMs

**Figure 3:**
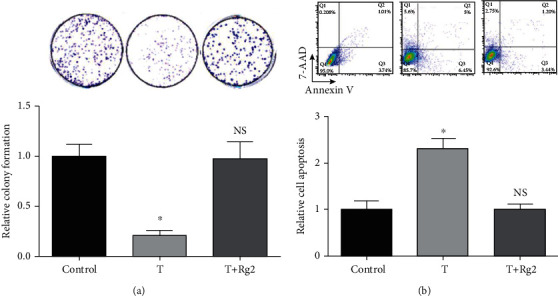
Rg2 promoted colony formation and inhibited TZM-induced apoptosis in HCMs: (a) colony formation ability of HCMs in three groups and (b) the apoptosis of HCMs in three groups. Data were shown as mean ± SD (*n* = 3). ^∗^*P* < 0.05 compared to other groups.

Next, we performed the colony formation assay and found that the colony formation ability of HCMs was significantly lower in the T group than in the control group but was recovered in the T+Rg2 group (Figure 3(a)). In contrast, apoptosis of HCMs was significantly higher in the T group than in the control group but was inhibited in the T+Rg2 group (Figure 3(b)). These results suggest that Rg2 promotes colony formation of HCMs and inhibits TZM-induced apoptosis in HCMs.

### 3.4. Rg2 Downregulated Proapoptotic Proteins in HCMs

**Figure 4:**
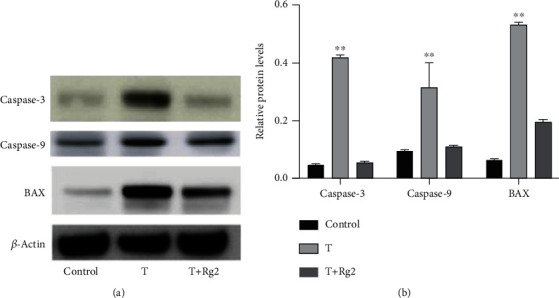
Rg2 downregulated proapoptotic proteins in HCMs: (a) representative blots of caspase-3, caspase-9, and BAX proteins in HCMs in three groups and (b) densitometry analysis of caspase-3, caspase-9, and BAX protein levels in HCMs in three groups. Data were shown as mean ± SD (*n* = 3). ^∗∗^*P* < 0.01 compared to other groups.

To understand how Rg2 inhibited TZM-induced apoptosis in HCMs, we focused on apoptosis-related proteins. Western blot analysis showed that caspase-3, caspase-9, and BAX protein levels were higher in the T group than in the control group but were lower after pretreatment with Rg2 (Figure 4(a)). Densitometry analysis showed that the differences in caspase-3, caspase-9, and BAX protein levels between T groups and control and T+Rg2 groups were significant (Figure 4(b)). These results suggest that Rg2 inhibits TZM-induced apoptosis in HCMs by downregulating the expression of proapoptotic proteins.

## 4. Discussion

In this study, we established a rat model of TZM-induced cardiotoxicity and investigated the efficacy of ginsenoside Rg2 on TZM-induced cardiotoxicity. We found that TZM induced LV dysfunction in rats, indicating the successful establishment of the model of TZM-induced cardiotoxicity. Based on this model, we performed echocardiography analysis and showed that Rg2 could attenuate TZM-induced LV dysfunction.

Mechanistically, we found that mRNA levels of caspase-3, caspase-9, and BAX were significantly higher in the rat model of TZM-induced cardiotoxicity based on RNAseq analysis. These results provide clue that TZM-induced LV dysfunction may be related to the induction of apoptosis of cardiomyocytes. The apoptosis of cardiomyocytes is known to be activated by oxidative stress [12, 13]. Interestingly, ginsenoside Rg2 has been reported to exhibit antioxidative effects and inhibit the apoptosis of different types of cells [14–16]. Therefore, we hypothesized that ginsenoside Rg2 may inhibit TZM-induced apoptosis of cardiomyocytes to reduce the cardiotoxicity of TZM.

To test our hypothesis, we employed HCMs as the experimental model. The colony formation assay showed that the colony formation ability of HCMs was inhibited by TZM but was recovered after pretreatment with Rg2. Consistently, apoptosis of HCMs was enhanced by TZM but was antagonized after pretreatment with Rg2. Furthermore, we detected apoptosis-related proteins and found that caspase-3, caspase-9, and BAX protein levels were upregulated by TZM but were downregulated after pretreatment with Rg2. Collectively, these results suggest that Rg2 abrogates TZM-induced apoptosis of HCMs by downregulating the expression of proapoptotic proteins such as caspase-3, caspase-9, and BAX.

An early study indicated that ginsenoside Rg2 could prevent the apoptosis of neurons via the phosphoinositide 3-kinase (PI3K)/Akt pathway [17]. In addition, a recent study showed that ginsenoside Rg2 promoted the activation of Akt in cardiomyocytes [18]. However, ginsenoside Rg2 activated the ROS-AMPK signaling pathway to inhibit the activation of ERK1/2 and Akt, leading to the inhibition of breast cancer cell proliferation and cell cycle progression and the induction of apoptosis [ 19, 20]. Therefore, the regulation of Akt by ginsenoside Rg2 may be dependent on different cell types. Further studies are needed to investigate whether ginsenoside Rg2 could activate the PI3K/Akt pathway to inhibit the apoptosis and promote the survival of HCMs.

In conclusion, we demonstrated that ginsenoside Rg2 inhibited TZM-induced cardiotoxicity and the mechanism may be related to the downregulation of the expression of proapoptotic proteins caspase-3, caspase-9, and BAX and the inhibition of TZM-induced apoptosis in cardiomyocytes. Ginsenoside Rg2 has a potential to be applied in patients with breast cancer to prevent TZM-induced cardiotoxicity.

## Data Availability

All data are available upon request.
